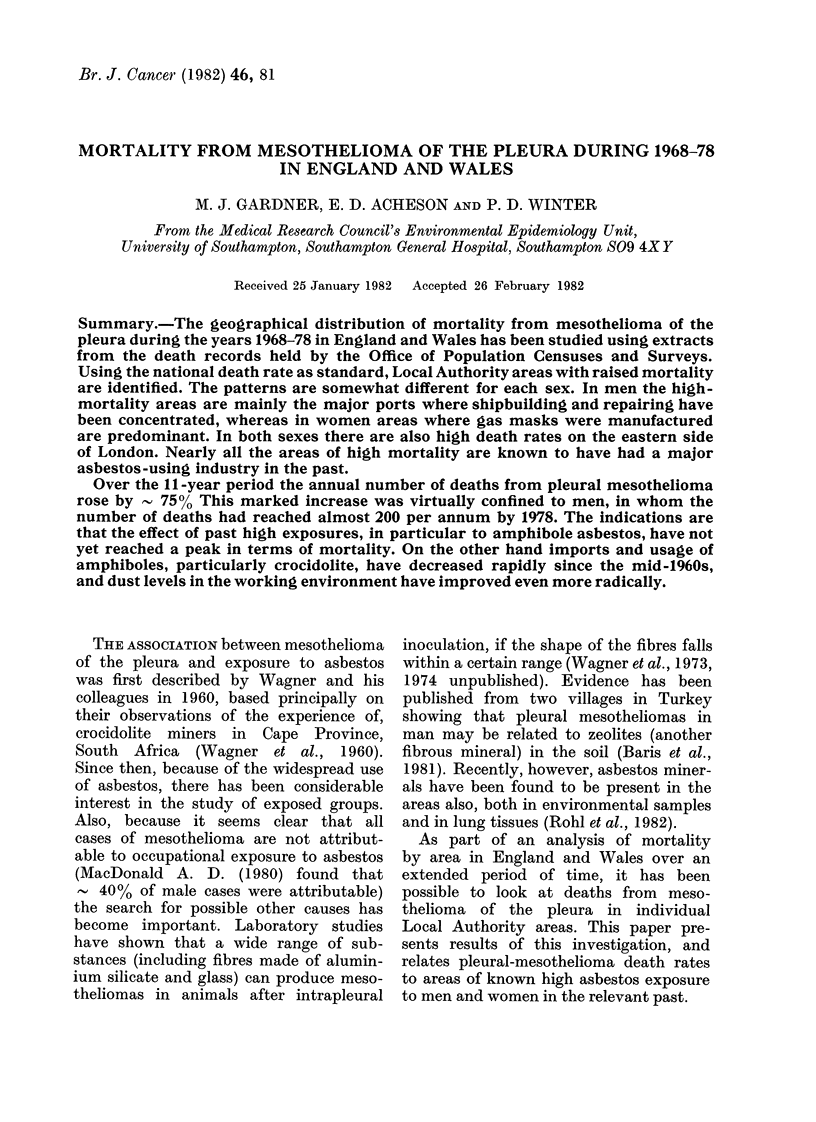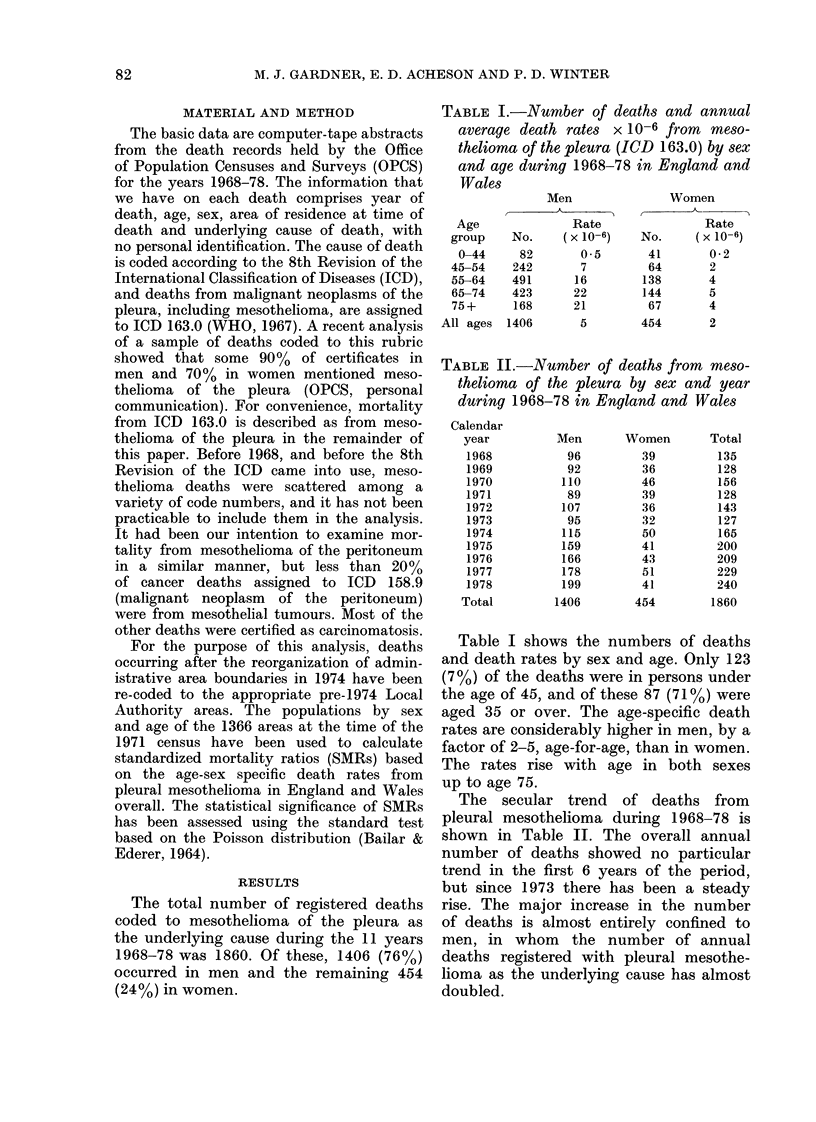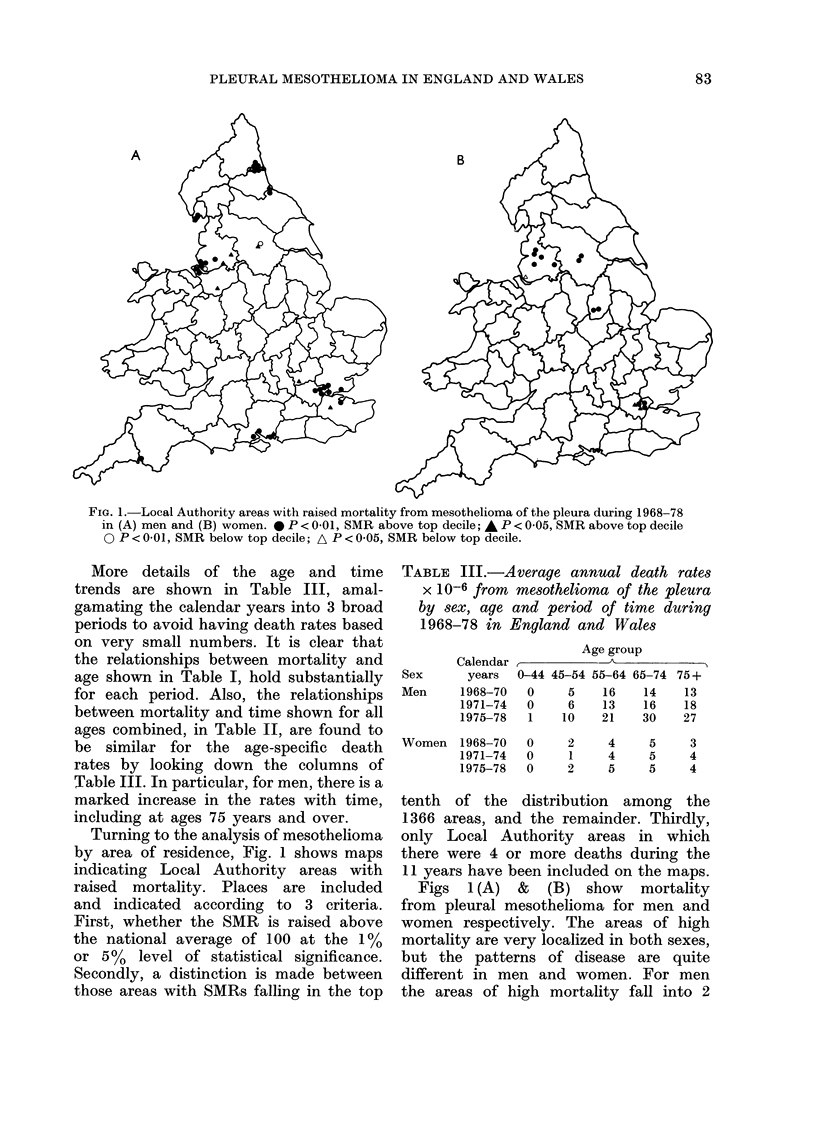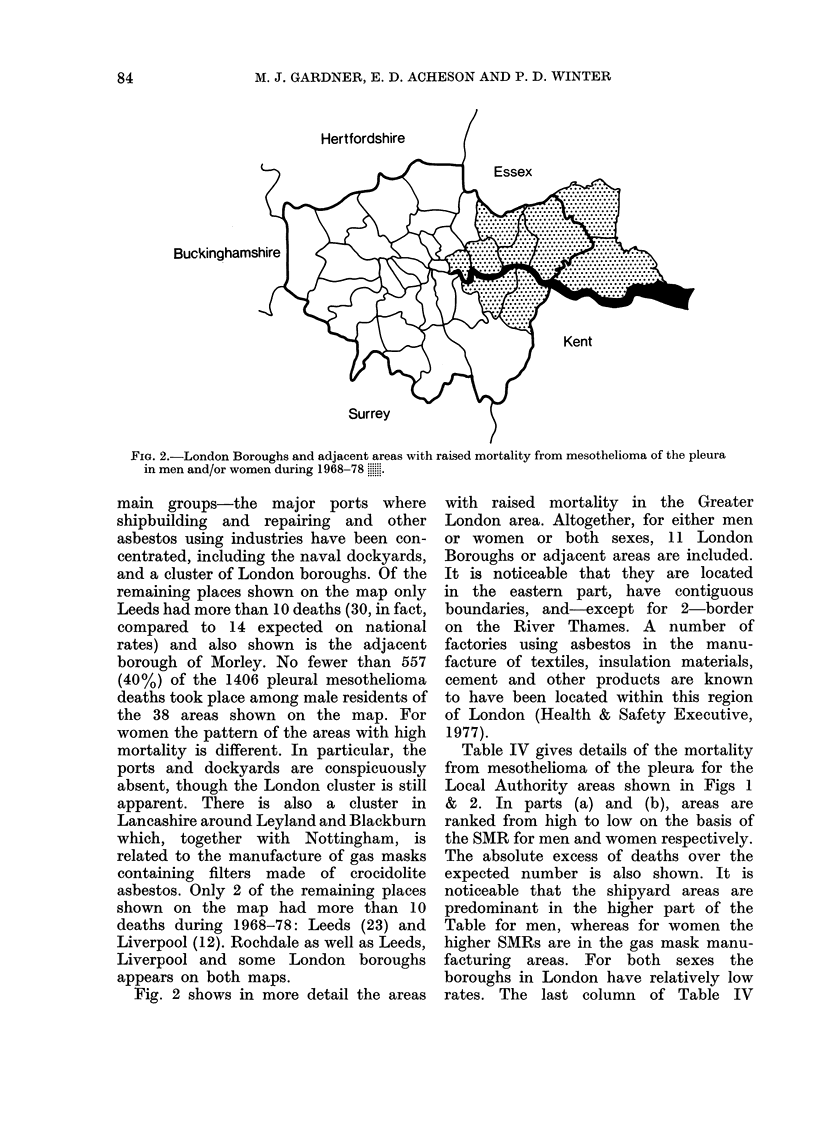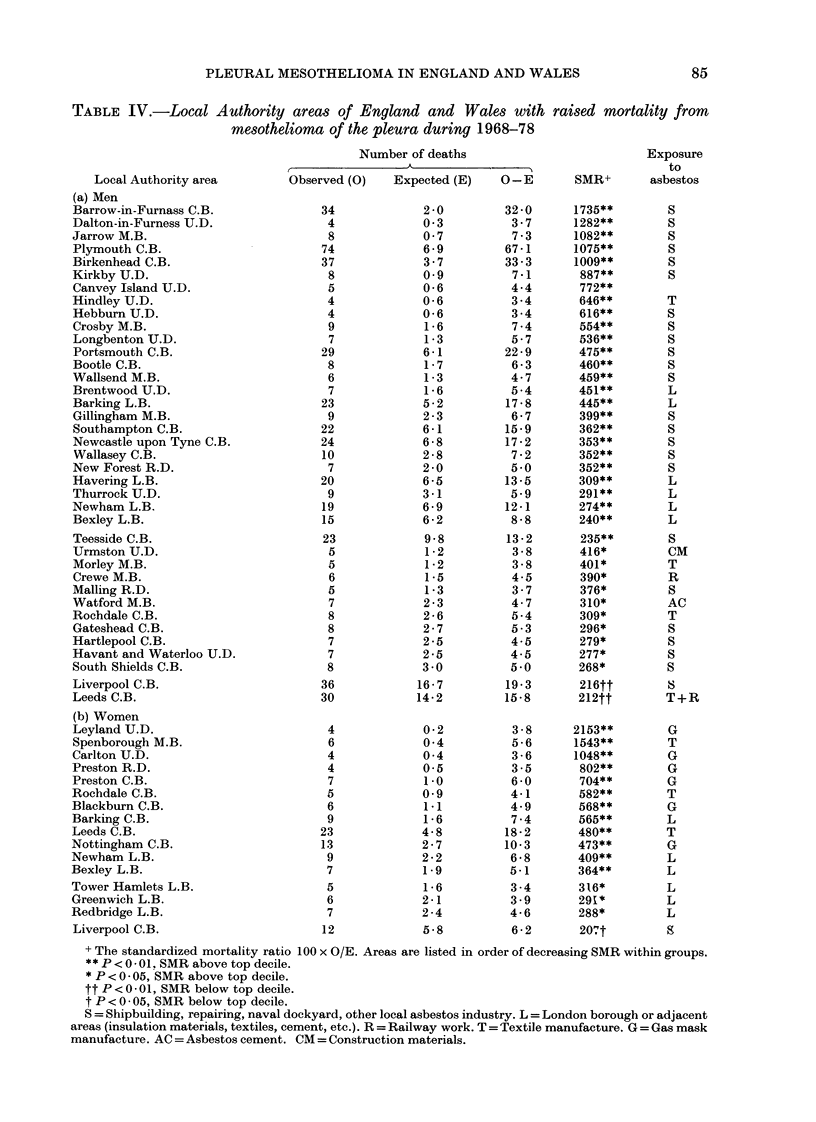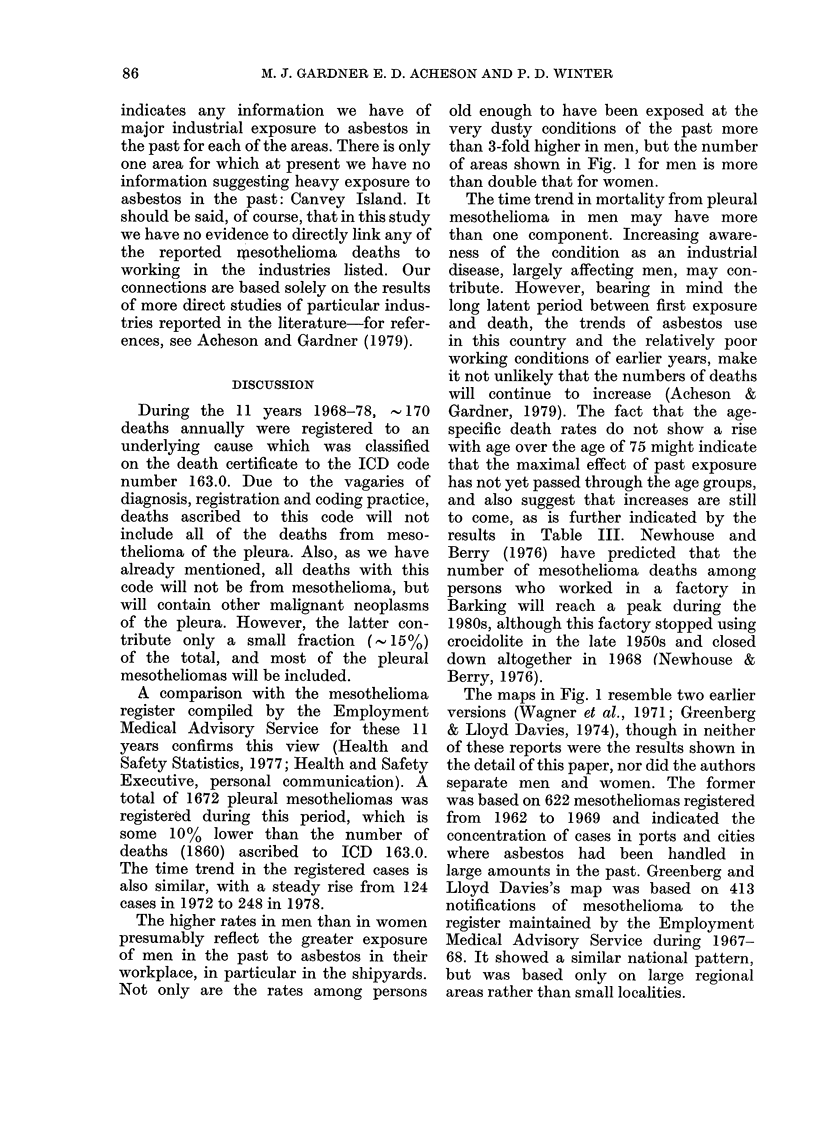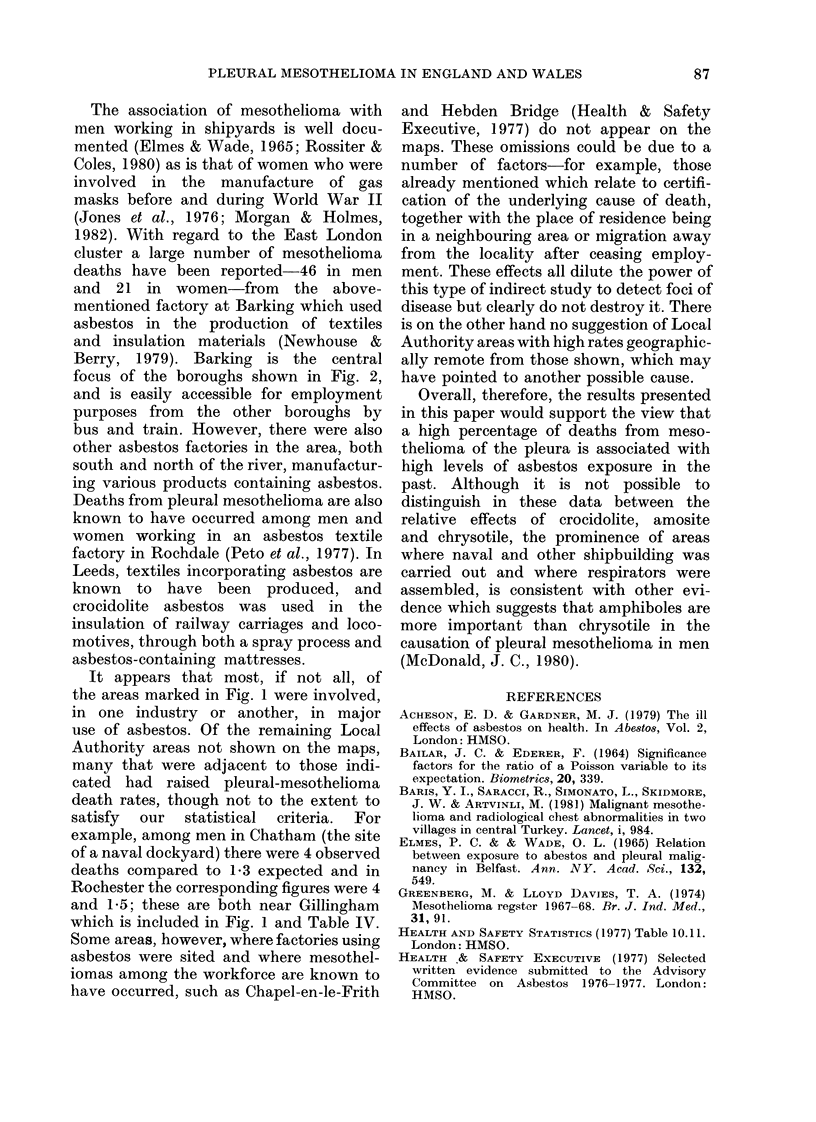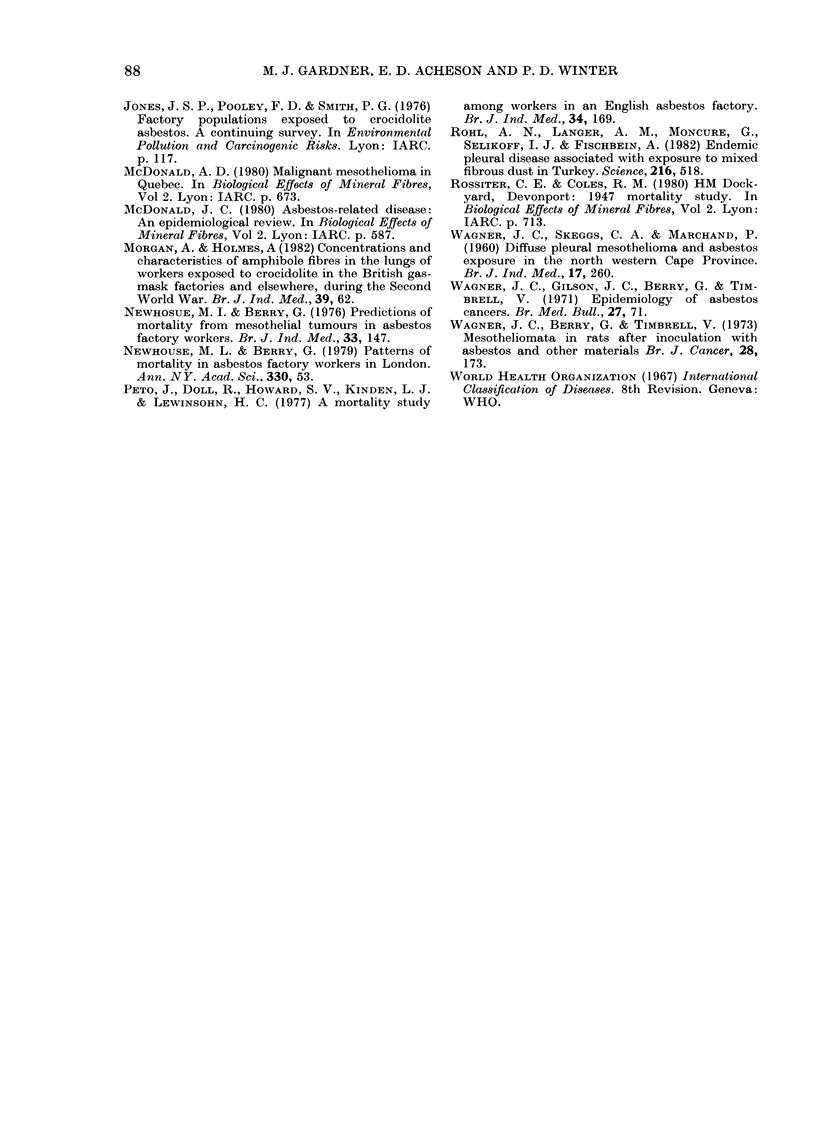# Mortality from mesothelioma of the pleura during 1968-78 in England and Wales.

**DOI:** 10.1038/bjc.1982.168

**Published:** 1982-07

**Authors:** M. J. Gardner, E. D. Acheson, P. D. Winter

## Abstract

The geographical distribution of mortality from mesothelioma of the pleura during the years 1968-78 in England and Wales has been studied using extracts from the death records held by the Office of Population Censuses and Surveys. Using the national death rate as standard, Local Authority areas with raised mortality are identified. The patterns are somewhat different for each sex. In men the high-mortality areas are mainly the major ports where shipbuilding and repairing have been concentrated, whereas in women areas where gas masks are manufactured are predominant. In both sexes there are also high death rates on the eastern side of London. Nearly all the areas of high mortality are known to have had a major asbestos-using industry in the past. Over the 11-year period the annual number of deaths from pleural mesothelioma rose by approximately 75%. This marked increase was virtually confined to men, in whom the number of deaths had reached almost 200 per annum by 1978. The indications are that the effect of past high exposures, in particular to amphibole asbestos, have not yet reached a peak in terms of mortality. On the other hand imports and usage of amphiboles, particularly crocidolite, have decreased rapidly since the mid-1960s, and dust levels in the working environment have improved even more radically.


*Br. J. Cancer* (1982) **46**, 81

### MORTALITY FROM MESOTHELIOMA OF THE PLEURA DURING 1968–78 IN ENGLAND AND WALES

M. J. GARDNER, E. D. ACHESON AND P. D. WINTER

*From the Medical Research Council's Environmental Epidemiology Unit, University of Southampton, Southampton General Hospital, Southampton SO9 4XY*

Received 25 January 1982 Accepted 26 February 1982

**Summary.—The geographical distribution of mortality from mesothelioma of the pleura during the years 1968–78 in England and Wales has been studied using extracts from the death records held by the Office of Population Censuses and Surveys. Using the national death rate as standard, Local Authority areas with raised mortality are identified. The patterns are somewhat different for each sex. In men the high-mortality areas are mainly the major ports where shipbuilding and repairing have been concentrated, whereas in women areas where gas masks were manufactured are predominant. In both sexes there are also high death rates on the eastern side of London. Nearly all the areas of high mortality are known to have had a major asbestos-using industry in the past.**

**Over the 11-year period the annual number of deaths from pleural mesothelioma rose by ~ 75% This marked increase was virtually confined to men, in whom the number of deaths had reached almost 200 per annum by 1978. The indications are that the effect of past high exposures, in particular to amphibole asbestos, have not yet reached a peak in terms of mortality. On the other hand imports and usage of amphiboles, particularly crocidolite, have decreased rapidly since the mid-1960s, and dust levels in the working environment have improved even more radically.**

THE ASSOCIATION between mesothelioma of the pleura and exposure to asbestos was first described by Wagner and his colleagues in 1960, based principally on their observations of the experience of, crocidolite miners in Cape Province, South Africa (Wagner *et al.*, 1960). Since then, because of the widespread use of asbestos, there has been considerable interest in the study of exposed groups. Also, because it seems clear that all cases of mesothelioma are not attributable to occupational exposure to asbestos (MacDonald A. D. (1980) found that ~ 40% of male cases were attributable) the search for possible other causes has become important. Laboratory studies have shown that a wide range of substances (including fibres made of aluminium silicate and glass) can produce mesotheliomas in animals after intrapleural inoculation, if the shape of the fibres falls within a certain range (Wagner *et al.*, 1973, 1974 unpublished). Evidence has been published from two villages in Turkey showing that pleural mesotheliomas in man may be related to zeolites (another fibrous mineral) in the soil (Baris *et al.*, 1981). Recently, however, asbestos minerals have been found to be present in the areas also, both in environmental samples and in lung tissues (Rohl *et al.*, 1982).

As part of an analysis of mortality by area in England and Wales over an extended period of time, it has been possible to look at deaths from mesothelioma of the pleura in individual Local Authority areas. This paper presents results of this investigation, and relates pleural-mesothelioma death rates to areas of known high asbestos exposure to men and women in the relevant past.

### MATERIAL AND METHOD

The basic data are computer-tape abstracts from the death records held by the Office of Population Censuses and Surveys (OPCS) for the years 1968–78. The information that we have on each death comprises year of death, age, sex, area of residence at time of death and underlying cause of death, with no personal identification. The cause of death is coded according to the 8th Revision of the International Classification of Diseases (ICD), and deaths from malignant neoplasms of the pleura, including mesothelioma, are assigned to ICD 163.0 (WHO, 1967). A recent analysis of a sample of deaths coded to this rubric showed that some 90% of certificates in men and 70% in women mentioned mesothelioma of the pleura (OPCS, personal communication). For convenience, mortality from ICD 163.0 is described as from mesothelioma of the pleura in the remainder of this paper. Before 1968, and before the 8th Revision of the ICD came into use, mesothelioma deaths were scattered among a variety of code numbers, and it has not been practicable to include them in the analysis. It had been our intention to examine mortality from mesothelioma of the peritoneum in a similar manner, but less than 20% of cancer deaths assigned to ICD 158.9 (malignant neoplasm of the peritoneum) were from mesothelial tumours. Most of the other deaths were certified as carcinomatosis.

For the purpose of this analysis, deaths occurring after the reorganization of administrative area boundaries in 1974 have been re-coded to the appropriate pre-1974 Local Authority areas. The populations by sex and age of the 1366 areas at the time of the 1971 census have been used to calculate standardized mortality ratios (SMRs) based on the age-sex specific death rates from pleural mesothelioma in England and Wales overall. The statistical significance of SMRs has been assessed using the standard test based on the Poisson distribution (Bailar & Ederer, 1964).

### RESULTS

The total number of registered deaths coded to mesothelioma of the pleura as the underlying cause during the 11 years 1968–78 was 1860. Of these, 1406 (76%) occurred in men and the remaining 454 (24%) in women.

TABLE I.—*Number of deaths and annual average death rates* $\times 10^{-6}$ *from mesothelioma of the pleura (ICD* 163.0) *by sex and age during* 1968–78 *in England and Wales*

| Age group | Men No. | Men Rate ($\times 10^{-6}$) | Women No. | Women Rate ($\times 10^{-6}$) |
|---|---|---|---|---|
| 0–44 | 82 | 0·5 | 41 | 0·2 |
| 45–54 | 242 | 7 | 64 | 2 |
| 55–64 | 491 | 16 | 138 | 4 |
| 65–74 | 423 | 22 | 144 | 5 |
| 75+ | 168 | 21 | 67 | 4 |
| All ages | 1406 | 5 | 454 | 2 |

TABLE II.—*Number of deaths from mesothelioma of the pleura by sex and year during* 1968–78 *in England and Wales*

| Calendar year | Men | Women | Total |
|---|---|---|---|
| 1968 | 96 | 39 | 135 |
| 1969 | 92 | 36 | 128 |
| 1970 | 110 | 46 | 156 |
| 1971 | 89 | 39 | 128 |
| 1972 | 107 | 36 | 143 |
| 1973 | 95 | 32 | 127 |
| 1974 | 115 | 50 | 165 |
| 1975 | 159 | 41 | 200 |
| 1976 | 166 | 43 | 209 |
| 1977 | 178 | 51 | 229 |
| 1978 | 199 | 41 | 240 |
| Total | 1406 | 454 | 1860 |

Table I shows the numbers of deaths and death rates by sex and age. Only 123 (7%) of the deaths were in persons under the age of 45, and of these 87 (71%) were aged 35 or over. The age-specific death rates are considerably higher in men, by a factor of 2–5, age-for-age, than in women. The rates rise with age in both sexes up to age 75.

The secular trend of deaths from pleural mesothelioma during 1968–78 is shown in Table II. The overall annual number of deaths showed no particular trend in the first 6 years of the period, but since 1973 there has been a steady rise. The major increase in the number of deaths is almost entirely confined to men, in whom the number of annual deaths registered with pleural mesothelioma as the underlying cause has almost doubled.

FIG. 1.—Local Authority areas with raised mortality from mesothelioma of the pleura during 1968–78 in (A) men and (B) women. ● $P<0{\cdot}01$, SMR above top decile; ▲ $P<0{\cdot}05$, SMR above top decile ○ $P<0{\cdot}01$, SMR below top decile; △ $P<0{\cdot}05$, SMR below top decile.

More details of the age and time trends are shown in Table III, amalgamating the calendar years into 3 broad periods to avoid having death rates based on very small numbers. It is clear that the relationships between mortality and age shown in Table I, hold substantially for each period. Also, the relationships between mortality and time shown for all ages combined, in Table II, are found to be similar for the age-specific death rates by looking down the columns of Table III. In particular, for men, there is a marked increase in the rates with time, including at ages 75 years and over.

Turning to the analysis of mesothelioma by area of residence, Fig. 1 shows maps indicating Local Authority areas with raised mortality. Places are included and indicated according to 3 criteria. First, whether the SMR is raised above the national average of 100 at the 1% or 5% level of statistical significance. Secondly, a distinction is made between those areas with SMRs falling in the top

TABLE III.—*Average annual death rates* $\times 10^{-6}$ *from mesothelioma of the pleura by sex, age and period of time during* 1968–78 *in England and Wales*

| Sex | Calendar years | Age group 0–44 | 45–54 | 55–64 | 65–74 | 75+ |
|---|---|---|---|---|---|---|
| Men | 1968–70 | 0 | 5 | 16 | 14 | 13 |
| | 1971–74 | 0 | 6 | 13 | 16 | 18 |
| | 1975–78 | 1 | 10 | 21 | 30 | 27 |
| Women | 1968–70 | 0 | 2 | 4 | 5 | 3 |
| | 1971–74 | 0 | 1 | 4 | 5 | 4 |
| | 1975–78 | 0 | 2 | 5 | 5 | 4 |

tenth of the distribution among the 1366 areas, and the remainder. Thirdly, only Local Authority areas in which there were 4 or more deaths during the 11 years have been included on the maps.

Figs 1(A) & (B) show mortality from pleural mesothelioma for men and women respectively. The areas of high mortality are very localized in both sexes, but the patterns of disease are quite different in men and women. For men the areas of high mortality fall into 2

FIG. 2.—London Boroughs and adjacent areas with raised mortality from mesothelioma of the pleura in men and/or women during 1968–78 ▒.

main groups—the major ports where shipbuilding and repairing and other asbestos using industries have been concentrated, including the naval dockyards, and a cluster of London boroughs. Of the remaining places shown on the map only Leeds had more than 10 deaths (30, in fact, compared to 14 expected on national rates) and also shown is the adjacent borough of Morley. No fewer than 557 (40%) of the 1406 pleural mesothelioma deaths took place among male residents of the 38 areas shown on the map. For women the pattern of the areas with high mortality is different. In particular, the ports and dockyards are conspicuously absent, though the London cluster is still apparent. There is also a cluster in Lancashire around Leyland and Blackburn which, together with Nottingham, is related to the manufacture of gas masks containing filters made of crocidolite asbestos. Only 2 of the remaining places shown on the map had more than 10 deaths during 1968–78: Leeds (23) and Liverpool (12). Rochdale as well as Leeds, Liverpool and some London boroughs appears on both maps.

Fig. 2 shows in more detail the areas with raised mortality in the Greater London area. Altogether, for either men or women or both sexes, 11 London Boroughs or adjacent areas are included. It is noticeable that they are located in the eastern part, have contiguous boundaries, and—except for 2—border on the River Thames. A number of factories using asbestos in the manufacture of textiles, insulation materials, cement and other products are known to have been located within this region of London (Health & Safety Executive, 1977).

Table IV gives details of the mortality from mesothelioma of the pleura for the Local Authority areas shown in Figs 1 & 2. In parts (a) and (b), areas are ranked from high to low on the basis of the SMR for men and women respectively. The absolute excess of deaths over the expected number is also shown. It is noticeable that the shipyard areas are predominant in the higher part of the Table for men, whereas for women the higher SMRs are in the gas mask manufacturing areas. For both sexes the boroughs in London have relatively low rates. The last column of Table IV

TABLE IV.—*Local Authority areas of England and Wales with raised mortality from mesothelioma of the pleura during* 1968–78

| Local Authority area | Number of deaths: Observed (O) | Expected (E) | O−E | SMR⁺ | Exposure to asbestos |
|---|---|---|---|---|---|
| (a) Men | | | | | |
| Barrow-in-Furnass C.B. | 34 | 2·0 | 32·0 | 1735** | S |
| Dalton-in-Furness U.D. | 4 | 0·3 | 3·7 | 1282** | S |
| Jarrow M.B. | 8 | 0·7 | 7·3 | 1082** | S |
| Plymouth C.B. | 74 | 6·9 | 67·1 | 1075** | S |
| Birkenhead C.B. | 37 | 3·7 | 33·3 | 1009** | S |
| Kirkby U.D. | 8 | 0·9 | 7·1 | 887** | S |
| Canvey Island U.D. | 5 | 0·6 | 4·4 | 772** | |
| Hindley U.D. | 4 | 0·6 | 3·4 | 646** | T |
| Hebburn U.D. | 4 | 0·6 | 3·4 | 616** | S |
| Crosby M.B. | 9 | 1·6 | 7·4 | 554** | S |
| Longbenton U.D. | 7 | 1·3 | 5·7 | 536** | S |
| Portsmouth C.B. | 29 | 6·1 | 22·9 | 475** | S |
| Bootle C.B. | 8 | 1·7 | 6·3 | 460** | S |
| Wallsend M.B. | 6 | 1·3 | 4·7 | 459** | S |
| Brentwood U.D. | 7 | 1·6 | 5·4 | 451** | L |
| Barking L.B. | 23 | 5·2 | 17·8 | 445** | L |
| Gillingham M.B. | 9 | 2·3 | 6·7 | 399** | S |
| Southampton C.B. | 22 | 6·1 | 15·9 | 362** | S |
| Newcastle upon Tyne C.B. | 24 | 6·8 | 17·2 | 353** | S |
| Wallasey C.B. | 10 | 2·8 | 7·2 | 352** | S |
| New Forest R.D. | 7 | 2·0 | 5·0 | 352** | S |
| Havering L.B. | 20 | 6·5 | 13·5 | 309** | L |
| Thurrock U.D. | 9 | 3·1 | 5·9 | 291** | L |
| Newham L.B. | 19 | 6·9 | 12·1 | 274** | L |
| Bexley L.B. | 15 | 6·2 | 8·8 | 240** | L |
| Teesside C.B. | 23 | 9·8 | 13·2 | 235** | S |
| Urmston U.D. | 5 | 1·2 | 3·8 | 416* | CM |
| Morley M.B. | 5 | 1·2 | 3·8 | 401* | T |
| Crewe M.B. | 6 | 1·5 | 4·5 | 390* | R |
| Malling R.D. | 5 | 1·3 | 3·7 | 376* | S |
| Watford M.B. | 7 | 2·3 | 4·7 | 310* | AC |
| Rochdale C.B. | 8 | 2·6 | 5·4 | 309* | T |
| Gateshead C.B. | 8 | 2·7 | 5·3 | 296* | S |
| Hartlepool C.B. | 7 | 2·5 | 4·5 | 279* | S |
| Havant and Waterloo U.D. | 7 | 2·5 | 4·5 | 277* | S |
| South Shields C.B. | 8 | 3·0 | 5·0 | 268* | S |
| Liverpool C.B. | 36 | 16·7 | 19·3 | 216†† | S |
| Leeds C.B. | 30 | 14·2 | 15·8 | 212†† | T+R |
| (b) Women | | | | | |
| Leyland U.D. | 4 | 0·2 | 3·8 | 2153** | G |
| Spenborough M.B. | 6 | 0·4 | 5·6 | 1543** | T |
| Carlton U.D. | 4 | 0·4 | 3·6 | 1048** | G |
| Preston R.D. | 4 | 0·5 | 3·5 | 802** | G |
| Preston C.B. | 7 | 1·0 | 6·0 | 704** | G |
| Rochdale C.B. | 5 | 0·9 | 4·1 | 582** | T |
| Blackburn C.B. | 6 | 1·1 | 4·9 | 568** | G |
| Barking C.B. | 9 | 1·6 | 7·4 | 565** | L |
| Leeds C.B. | 23 | 4·8 | 18·2 | 480** | T |
| Nottingham C.B. | 13 | 2·7 | 10·3 | 473** | G |
| Newham L.B. | 9 | 2·2 | 6·8 | 409** | L |
| Bexley L.B. | 7 | 1·9 | 5·1 | 364** | L |
| Tower Hamlets L.B. | 5 | 1·6 | 3·4 | 316* | L |
| Greenwich L.B. | 6 | 2·1 | 3·9 | 291* | L |
| Redbridge L.B. | 7 | 2·4 | 4·6 | 288* | L |
| Liverpool C.B. | 12 | 5·8 | 6·2 | 207† | S |

⁺ The standardized mortality ratio 100 × O/E. Areas are listed in order of decreasing SMR within groups.
** $P < 0\cdot01$, SMR above top decile.
* $P < 0\cdot05$, SMR above top decile.
†† $P < 0\cdot01$, SMR below top decile.
† $P < 0\cdot05$, SMR below top decile.
S = Shipbuilding, repairing, naval dockyard, other local asbestos industry. L = London borough or adjacent areas (insulation materials, textiles, cement, etc.). R = Railway work. T = Textile manufacture. G = Gas mask manufacture. AC = Asbestos cement. CM = Construction materials.

indicates any information we have of major industrial exposure to asbestos in the past for each of the areas. There is only one area for which at present we have no information suggesting heavy exposure to asbestos in the past: Canvey Island. It should be said, of course, that in this study we have no evidence to directly link any of the reported mesothelioma deaths to working in the industries listed. Our connections are based solely on the results of more direct studies of particular industries reported in the literature—for references, see Acheson and Gardner (1979).

### DISCUSSION

During the 11 years 1968–78, ~170 deaths annually were registered to an underlying cause which was classified on the death certificate to the ICD code number 163.0. Due to the vagaries of diagnosis, registration and coding practice, deaths ascribed to this code will not include all of the deaths from mesothelioma of the pleura. Also, as we have already mentioned, all deaths with this code will not be from mesothelioma, but will contain other malignant neoplasms of the pleura. However, the latter contribute only a small fraction (~15%) of the total, and most of the pleural mesotheliomas will be included.

A comparison with the mesothelioma register compiled by the Employment Medical Advisory Service for these 11 years confirms this view (Health and Safety Statistics, 1977; Health and Safety Executive, personal communication). A total of 1672 pleural mesotheliomas was registered during this period, which is some 10% lower than the number of deaths (1860) ascribed to ICD 163.0. The time trend in the registered cases is also similar, with a steady rise from 124 cases in 1972 to 248 in 1978.

The higher rates in men than in women presumably reflect the greater exposure of men in the past to asbestos in their workplace, in particular in the shipyards. Not only are the rates among persons old enough to have been exposed at the very dusty conditions of the past more than 3-fold higher in men, but the number of areas shown in Fig. 1 for men is more than double that for women.

The time trend in mortality from pleural mesothelioma in men may have more than one component. Increasing awareness of the condition as an industrial disease, largely affecting men, may contribute. However, bearing in mind the long latent period between first exposure and death, the trends of asbestos use in this country and the relatively poor working conditions of earlier years, make it not unlikely that the numbers of deaths will continue to increase (Acheson & Gardner, 1979). The fact that the age-specific death rates do not show a rise with age over the age of 75 might indicate that the maximal effect of past exposure has not yet passed through the age groups, and also suggest that increases are still to come, as is further indicated by the results in Table III. Newhouse and Berry (1976) have predicted that the number of mesothelioma deaths among persons who worked in a factory in Barking will reach a peak during the 1980s, although this factory stopped using crocidolite in the late 1950s and closed down altogether in 1968 (Newhouse & Berry, 1976).

The maps in Fig. 1 resemble two earlier versions (Wagner *et al.*, 1971; Greenberg & Lloyd Davies, 1974), though in neither of these reports were the results shown in the detail of this paper, nor did the authors separate men and women. The former was based on 622 mesotheliomas registered from 1962 to 1969 and indicated the concentration of cases in ports and cities where asbestos had been handled in large amounts in the past. Greenberg and Lloyd Davies's map was based on 413 notifications of mesothelioma to the register maintained by the Employment Medical Advisory Service during 1967–68. It showed a similar national pattern, but was based only on large regional areas rather than small localities.

The association of mesothelioma with men working in shipyards is well documented (Elmes & Wade, 1965; Rossiter & Coles, 1980) as is that of women who were involved in the manufacture of gas masks before and during World War II (Jones *et al.*, 1976; Morgan & Holmes, 1982). With regard to the East London cluster a large number of mesothelioma deaths have been reported—46 in men and 21 in women—from the abovementioned factory at Barking which used asbestos in the production of textiles and insulation materials (Newhouse & Berry, 1979). Barking is the central focus of the boroughs shown in Fig. 2, and is easily accessible for employment purposes from the other boroughs by bus and train. However, there were also other asbestos factories in the area, both south and north of the river, manufacturing various products containing asbestos. Deaths from pleural mesothelioma are also known to have occurred among men and women working in an asbestos textile factory in Rochdale (Peto *et al.*, 1977). In Leeds, textiles incorporating asbestos are known to have been produced, and crocidolite asbestos was used in the insulation of railway carriages and locomotives, through both a spray process and asbestos-containing mattresses.

It appears that most, if not all, of the areas marked in Fig. 1 were involved, in one industry or another, in major use of asbestos. Of the remaining Local Authority areas not shown on the maps, many that were adjacent to those indicated had raised pleural-mesothelioma death rates, though not to the extent to satisfy our statistical criteria. For example, among men in Chatham (the site of a naval dockyard) there were 4 observed deaths compared to 1·3 expected and in Rochester the corresponding figures were 4 and 1·5; these are both near Gillingham which is included in Fig. 1 and Table IV. Some areas, however, where factories using asbestos were sited and where mesotheliomas among the workforce are known to have occurred, such as Chapel-en-le-Frith and Hebden Bridge (Health & Safety Executive, 1977) do not appear on the maps. These omissions could be due to a number of factors—for example, those already mentioned which relate to certification of the underlying cause of death, together with the place of residence being in a neighbouring area or migration away from the locality after ceasing employment. These effects all dilute the power of this type of indirect study to detect foci of disease but clearly do not destroy it. There is on the other hand no suggestion of Local Authority areas with high rates geographically remote from those shown, which may have pointed to another possible cause.

Overall, therefore, the results presented in this paper would support the view that a high percentage of deaths from mesothelioma of the pleura is associated with high levels of asbestos exposure in the past. Although it is not possible to distinguish in these data between the relative effects of crocidolite, amosite and chrysotile, the prominence of areas where naval and other shipbuilding was carried out and where respirators were assembled, is consistent with other evidence which suggests that amphiboles are more important than chrysotile in the causation of pleural mesothelioma in men (McDonald, J. C., 1980).

### REFERENCES

ACHESON, E. D. & GARDNER, M. J. (1979) The ill effects of asbestos on health. In *Abestos*, Vol. 2, London: HMSO.

BAILAR, J. C. & EDERER, F. (1964) Significance factors for the ratio of a Poisson variable to its expectation. *Biometrics*, **20**, 339.

BARIS, Y. I., SARACCI, R., SIMONATO, L., SKIDMORE, J. W. & ARTVINLI, M. (1981) Malignant mesothelioma and radiological chest abnormalities in two villages in central Turkey. *Lancet*, i, 984.

ELMES, P. C. & & WADE, O. L. (1965) Relation between exposure to abestos and pleural malignancy in Belfast. *Ann. NY. Acad. Sci.*, **132**, 549.

GREENBERG, M. & LLOYD DAVIES, T. A. (1974) Mesothelioma regster 1967–68. *Br. J. Ind. Med.*, **31**, 91.

HEALTH AND SAFETY STATISTICS (1977) Table 10.11. London: HMSO.

HEALTH & SAFETY EXECUTIVE (1977) Selected written evidence submitted to the Advisory Committee on Asbestos 1976–1977. London: HMSO.

JONES, J. S. P., POOLEY, F. D. & SMITH, P. G. (1976) Factory populations exposed to crocidolite asbestos. A continuing survey. In *Environmental Pollution and Carcinogenic Risks*. Lyon: IARC. p. 117.

MCDONALD, A. D. (1980) Malignant mesothelioma in Quebec. In *Biological Effects of Mineral Fibres*, Vol 2. Lyon: IARC. p. 673.

MCDONALD, J. C. (1980) Asbestos-related disease: An epidemiological review. In *Biological Effects of Mineral Fibres*, Vol 2. Lyon: IARC. p. 587.

MORGAN, A. & HOLMES, A (1982) Concentrations and characteristics of amphibole fibres in the lungs of workers exposed to crocidolite in the British gas-mask factories and elsewhere, during the Second World War. *Br. J. Ind. Med.*, **39**, 62.

NEWHOSUE, M. I. & BERRY, G. (1976) Predictions of mortality from mesothelial tumours in asbestos factory workers. *Br. J. Ind. Med.*, **33**, 147.

NEWHOUSE, M. L. & BERRY, G. (1979) Patterns of mortality in asbestos factory workers in London. *Ann. NY. Acad. Sci.*, **330**, 53.

PETO, J., DOLL, R., HOWARD, S. V., KINDEN, L. J. & LEWINSOHN, H. C. (1977) A mortality study among workers in an English asbestos factory. *Br. J. Ind. Med.*, **34**, 169.

ROHL, A. N., LANGER, A. M., MONCURE, G., SELIKOFF, I. J. & FISCHBEIN, A. (1982) Endemic pleural disease associated with exposure to mixed fibrous dust in Turkey. *Science*, **216**, 518.

ROSSITER, C. E. & COLES, R. M. (1980) HM Dockyard, Devonport: 1947 mortality study. In *Biological Effects of Mineral Fibres*, Vol 2. Lyon: IARC. p. 713.

WAGNER, J. C., SKEGGS, C. A. & MARCHAND, P. (1960) Diffuse pleural mesothelioma and asbestos exposure in the north western Cape Province. *Br. J. Ind. Med.*, **17**, 260.

WAGNER, J. C., GILSON, J. C., BERRY, G. & TIMBRELL, V. (1971) Epidemiology of asbestos cancers. *Br. Med. Bull.*, **27**, 71.

WAGNER, J. C., BERRY, G. & TIMBRELL, V. (1973) Mesotheliomata in rats after inoculation with asbestos and other materials *Br. J. Cancer*, **28**, 173.

WORLD HEALTH ORGANIZATION (1967) *International Classification of Diseases*. 8th Revision. Geneva: WHO.